# An alternative asymmetric figure-of-eight single-layer suture technique for bowel anastomosis in an *in vitro* porcine model

**DOI:** 10.3389/fsurg.2022.896542

**Published:** 2022-09-28

**Authors:** Chen Liu, Yewen Wang, Ai-rong Zhao, Feng-ai Hu, Qizhong Fan, Guoxiu Han, Guojian Ding, Tingliang Fu, Lei Geng, Hongshan Yin

**Affiliations:** ^1^ The Department of Pediatric Surgery, Binzhou Medical University Hospital, Binzhou, China; ^2^ The Department of Surgery, Shanghai Children's Hospital, Shanghai Jiao Tong University, Shanghai, China; ^3^ The Department of Pharmacy, Binzhou Medical University Hospital, Binzhou, China; ^4^ The Clinical Laboratory, Binzhou Medical University Hospital, Binzhou, China; ^5^ The Department of Urology, Binzhou Medical University Hospital, Binzhou, China

**Keywords:** intestinal anastomosis, single-layer suture, asymmetric figure-of-eight suture, anastomotic leakage, *in vitro* experiment

## Abstract

Anastomotic techniques are of vital importance in restoring gastrointestinal continuity after resection. An alternative asymmetric figure-of-eight single-layer suture anastomotic technique was introduced and its effects were evaluated in an *in vitro* porcine model. Twelve 15-cm grossly healthy small intestine segments from a porcine cadaver were harvested and randomly divided into asymmetric figure-of-eight single-layer suture (figure-of-eight suture) and single-layer interrupted suture technique (interrupted suture) groups (*n* = 6 in each group). The anastomosed bowel was infused with methylene blue solution to test anastomotic leakage. Anastomosis construction time, leakage, and suture material cost were recorded and analyzed statistically using Fisher's exact test and Student's *t*-test. One anastomotic leakage occurred (16.67%) in the figure-of-eight suture group, and two (33.33%) in the interrupted suture group (*p* > 0.9999). The anastomosis construction time was relatively short in the figure-of-eight suture group, but the difference did not reach a statistically significant level between the two groups. The mean number of suture knots and the cost of suture material in the figure-of-eight suture group were significantly decreased in comparison to the interrupted suture group (15.67 ± 3.30 vs. 22.17 ± 2.03, 167.11 ± 35.20 vs. 236.45 ± 21.70 CNY, *p* < 0.01, respectively). Our results suggested that the alternative asymmetric figure-of-eight suture technique was safe and economic for intestinal anastomosis. An *in vivo* experiment is required to elucidate the effects of this suture technique on the physiological anastomotic healing process.

## Introduction

Intestinal anastomosis is an essential operative procedure in restoring the continuity of the gastrointestinal tract after resection of bowel lesions, such as necrotic intestinal segments, malignancy, inflammatory bowel disease, and trauma (1–4). In comparison with adult surgery, small intestine anastomosis is difficult and has a high risk of complications due to the smaller diameter of the intestine, inflammation, edema, or immaturity in the pediatric group, especially in premature infants (5–7).

To effectively reduce the occurrence of complications related to anastomosis and to improve patients' surgical outcomes, various modified anastomosis techniques have been developed (8, 9). Double-layer anastomosis may lead to increased tissue damage and impaired blood supply with possibly delayed healing. Single-layer anastomosis is proved to be effective and safe (10, 11). The drawback of hand-sewn single-layer anastomosis is its insecurity in case of high-risk intestinal anastomosis, such as an edematous intestine (11). When the stitches are tied, excessive tension placed on the suture might result in tissue cutting, even anastomotic leakage (12, 13). In the 1950s, Gambee (14) designed a single-layer anastomosis technique. After that, the Gambee stitch and its modification procedures have been widely accepted in gastrointestinal anastomosis (15–17). As an advantage, this technique can improve tissue healing because of minimal disturbance in the blood supply of the anastomotic site (14, 17). However, the Gambee pattern requires more needle manipulation, a modified Gambee stitch that includes the second stitch (through the mucosa on the opposite side to the submucosa), and the third stitch (through the submucosa to the mucosa of the first side) was described by Shureih et al. (16). This technique requires less needle manipulation, being easier to perform with the same good results. Gambee et al. (17) reported subsequent experiences, including the posterior half of the anastomosis sutured within the lumen and the anterior half sutured by the classic Gambee stitch. Its advantage is easy to perform. Liang (18) introduced another single-layer suture technique with simplicity and reliability.

Previous studies revealed that the single-layer continuous Lembert pattern anastomosis is faster to perform and as strong as a two-layer anastomosis (9, 10, 19). Slieker et al. (20) analyzed the literature and concluded that a continuous suture is preferable for completing an anastomosis owing to the technical and time-consuming nature, although clinical and experimental studies have not revealed that the continuous suture technique is superior to the interrupted one. Modified Gambee stitch should remain an option in bowel anastomosis (2). Herein, we describe an alternative asymmetric figure-of-eight single-layer suture method, aiming to create a model that is safe, feasible, and easy to perform. Its feasibility and effectiveness were evaluated in an *in vitro* porcine anastomosis model.

## Materials and methods

### Study design

The research was an *in vitro* experiment on small intestine segments harvested from a healthy pig sacrificed at an abattoir. The effects of the figure-of-eight suture technique were compared with those of the interrupted suture technique. The study was approved by the Institutional Ethics Committee of Binzhou Medical University Hospital (No. 20210120-01), Shandong Province, China. No live animal was involved in the present study.

### Procedures

#### Small intestine harvest and preservation

The ileum of 180 cm in length was immediately harvested after the healthy pig was sacrificed by electrocution at an abattoir as described in the literature (21, 22). No gross abnormal appearance was observed during the porcine intestine preparation. The specimen was placed in an aseptic plastic bag within a box filled with iced water and was transported to the laboratory and placed in cold normal saline solution with 0.5% povidone-iodine solution (23). Luminal contents were gently milked and then flushed with cold normal saline solution. Ileum transection, anastomosis, and tests were completed within 9 h after the harvest of the specimen, as described by other authors (24, 25).

#### Sampling and groups

Based on the literature review on experimental intestinal anastomosis techniques, several studies have employed a sample size of six bowel segments per group (23, 26–29). Accordingly, a sample size of six in each group was determined in the present study.

The intestinal segment was then separated into 12 smaller segments (15 cm each), which were randomly divided into figure-of-eight and interrupted suture groups using the random number table. In the middle part of each segment, transection was conducted and anastomoses were then performed using a 4-0 polyglycolic acid suture with an atraumatic taper point needle. Both figure-of-eight and interrupted suture techniques were performed by the same senior surgeon, representing a homogeneous group of anastomoses.

#### Single-layer asymmetric figure-of-eight suture

**Figure 1:**
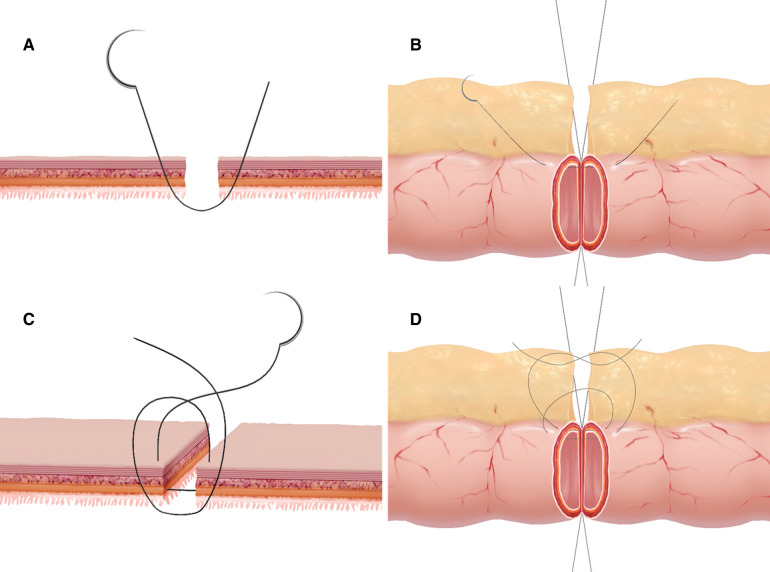
Graphic representation of the asymmetric figure-of-eight suture technic. (**A,B**) The first insertion and withdrawal of the needle was performed according to the sequence of "serosa, muscular, submucosa, and mucosa to contralateral mucosa, submucosa, muscular, and serosa" to sew more seromuscular and submucosal layers and less mucosal layer. (**C,D**) The second insertion and withdrawal of the needle was performed according to the sequence of "serosa, muscular, and submucosa to contralateral submucosa, muscular, and serosa" by taking a bite 1 mm from the cutting edge and forward from the first insertion level.

This alternative technique involved inserting and pulling out the needle twice in different planes. The steps of the single-layer asymmetric figure-of-eight suture were described as follows. Two 5/0 silk stay sutures were placed at the mesenteric and antimesenteric margins to bring the two intestinal ends together. Thereafter, the anastomosis was performed at the anterior wall with interrupted 4/0 absorbable sutures and the first point was placed on the mesenteric border. The first insertion and withdrawal of the needle were implemented by taking a bite of 2 mm from the wound edge. The needle was obliquely inserted into the serosa, muscularis, and submucosa and then pulled out 1 mm from the mucosa wound edge. Then, taking a bite 1 mm from the wound edge of the contralateral mucosal layer was made and directed obliquely through the submucosa, muscularis, and serosa 2 mm away from the wound edge (3 mm from the wound edge at the mesenteric site). The second insertion from the serosa and pulling out the needle from the submucosa, then from the submucosa through the serosa was taken at a point 1 mm from the edge (2 mm at the mesenteric site) and 1 mm forward from the first stitch plane, making the two stitches at different horizontal levels. The suture was tied on the serosal surface using three square knots with proper strength to avoid bowel strangulation. When the whole anterior wall was anastomosed, the posterior wall was sutured in the same way as described in the literature (30). The anastomotic technique was shown in Figure 1. The mesentery at the anastomosis site was dissected only 3 mm on each side of the bowel wound edge to make a good exposure and to ensure adequate approximation after completion of the anastomosis.

#### Single-layer interrupted suture anastomosis

The single-layer interrupted suture technique was conducted 2 mm from the wound edge and 2 mm apart between the stitches (3 mm from the wound edge at the mesenteric site).

#### Anastomosis construction time

The time (in minutes) for the creation of an anastomosis was recorded from the first suture bite to the completion of the anastomosis.

#### Leakage testing

**Figure 2:**
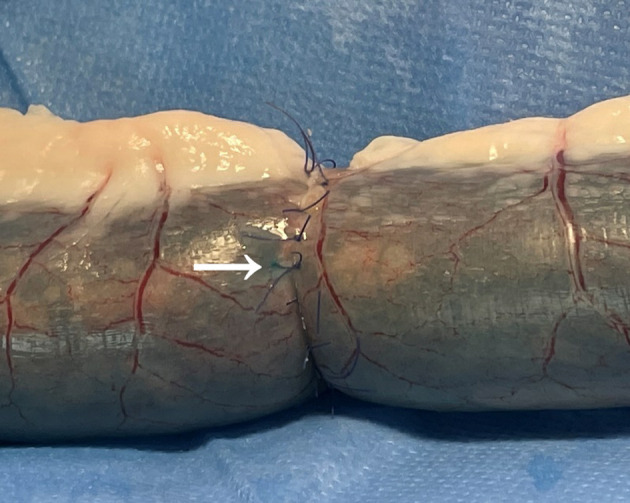
Leakage pressure testing showed the leak site with a suture hole (arrow).

After anastomosis completion, the specimen was stored in normal saline solution at room temperature and tested within 1 h. The leakage testing was conducted essentially as described previously (23, 31–36). Briefly, a 4-cm flexible pipe was tightly connected to a 50-ml syringe, which was inserted into the proximal end of the anastomosis. A hard plastic conduit, which was inserted into the distal end of the bowel segment, was connected to the pressure manometer. Both ends of the bowel segment with one pipe and one conduit were tightly ligated using a self-made blocking band. A digital pressure manometer (Xuzhou Engel Electronics Engineering Company, China) was used for monitoring the intraluminal pressure. The maximal intraluminal pressure was set at 30 mmHg as described in the literature (32)*.* Methylene blue was mixed with normal saline at 1:250 dilution. When the intraluminal pressure reached 30 mmHg, all specimens were observed by one investigator for leakage and leakage sites (anastomosis line or suture hole), as shown in Figure 2. The intraluminal pressure was continuously recorded using a digital camera.

#### The number of suture knots and cost of suture materials

The number of suture knots represented the number of sutures used on each anastomosis by simulating the clinical scenario in which sutures cannot be reused in the intraoperative setting. The number of suture knots of the anastomosis multiplied by 10.67 (the price of each suture in Chinese Yuan, CNY) was the cost of suture material.

#### Statistical methods

All continuous data were analyzed using Student's *t*-test and the results were expressed as the mean ± standard derivation. Categorical data were analyzed using Fisher's exact test. The anastomosis construction time, the number of suture knots, and suture material cost were compared using a paired Student's *t*-test. Fisher's exact test was used to compare the occurrence of anastomotic leakage at an intraluminal pressure of 30 mmHg. All statistical analyses were performed using GraphPad Prism version 8 (GraphPad Software Inc., San Diego, CA, USA) and IBM SPSS Statistics software version 25 (IBM Corp., Armonk, NY, USA). The level of statistical significance was set at *p* < 0.05.

## Results

The anastomosis construction time was 18.08 ± 5.43 min in the figure-of-eight suture group and 19.95 ± 5.21 min) as shown in Table 1. The mean time of anastomosis construction was relatively short in the figure-of-eight suture group, although a significant difference could not be reached between the two groups.

**Table 1:** Variables of the figure-of-eight and the interrupted suture groups in a porcine model with averages and standard deviations.

Variables	Figure-of-eight	Interrupted	*p*-value
	*n* = 6	*n* = 6	
Construction time (minutes)	18.08 ± 5.43	19.95 ± 5.21	0.5913^a^
The number of suture knots	15.67 ± 3.30	22.17 ± 2.03	0.0038^a^
Cost of suture materials (CNY)	167.11 ± 35.20	236.45 ± 21.70	0.0038^a^

^a^ Student's t-test.

When the intraluminal pressure reached 30 mmHg, one (16.67%) leakage at the anastomotic line occurred in the figure-of-eight suture group (24 mmHg), while two (33.33%) leakages (at the anastomotic line and suture hole at 24 and 28 mmHg, respectively) in the interrupted suture group. No significant difference was noted between the two groups (*p* > 0.9999).

The suture knots in the figure-of-eight suture group were 15.67 ± 3.30, which were significantly reduced in comparison with the interrupted suture group (22.17 ± 2.03; *p* = 0.0038). The mean cost of suture material was significantly decreased in the figure-of-eight suture group than that in the interrupted suture group (167.11 ± 35.20 vs. 236.45 ± 21.70 CNY, *p* = 0.0038), as shown in Table 1.

**Figure 3:**
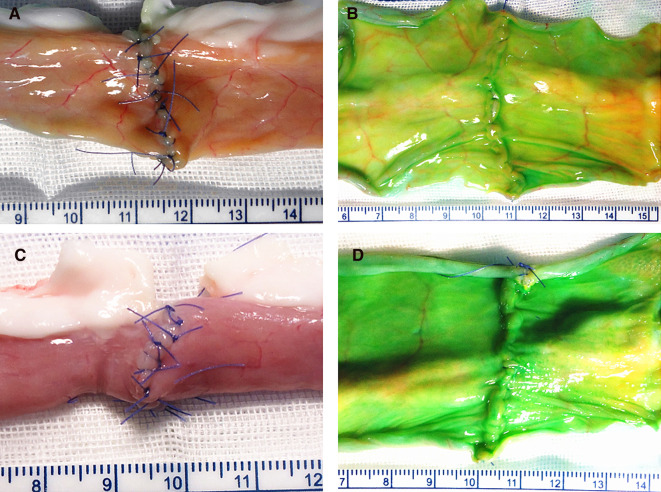
Extra- and intraluminal appearances of the two techniques: figure-of-eight suture technique (**A,B**); interrupted suture technique (**C,D**).

The extra- and intraluminal appearances of the two different anastomosis techniques were all tightly approximations, as shown in Figure 3.

## Discussion

Intestinal anastomosis for restoring gut continuity is an essential procedure (1–4). Owing to the smaller diameter and immature intestine in small infants and neonates, intestinal anastomosis remains a challenge for surgeons. The single-layer intestinal anastomosis has been proven safe and effective. However, its insecurity in case of high-risk intestinal anastomoses, such as an edematous intestine or severe bowel inflammation, might lead to a change of intraoperative strategy (5, 7, 11, 19). Based on intestinal anastomosis facileness and biomechanics, we designed an alternative asymmetric figure-of-eight suture and evaluated its effects through *in vitro* experiments on a porcine ileum specimen. Fresh bowel specimens were cleaned, manipulated, and monitored within 9 h after animal sacrifice to minimize the impact of the storage duration on the results (25, 37).

In the present study, the results showed that the asymmetric figure-of-eight suture required fewer suture knots in the anastomosis, which should save time by lesser needle manipulation and knot tying, although the time difference did not reach a statistically significant level owing to the small sample size. Rapid completion of an anastomosis with minimal trauma to the bowel is an important determinant of success in intestinal anastomosis, especially in those subjects that are suffering from a life-threatening gastrointestinal problem related to serious systemic condition, such as hemodynamic instability (38).

Anastomotic leakage is a severe postoperative complication that may lead to diffuse peritonitis, sepsis, and even life-threatening conditions (37, 39). Studies, including techniques, materials, and perioperative care have aimed to improve the outcome of intestinal anastomosis (20, 31, 40, 41). Intraoperative detection of the anastomosis quality and repairing defects without delay can significantly reduce the risk of postoperative leakage (31, 42).

Air testing, methylene blue perfusion testing, and intraoperative colonoscopy were selected to detect anastomotic leaks in both the *in vitro* experimental model and clinical research (2, 31, 42). In the present study, intraluminal leakage pressure with methylene blue perfusion was conducted to detect any potential anastomotic leakage as described by the previous study (42). Roumen et al. (32) reported that no adverse effects on the anastomosis occurred with the intraluminal pressure set at 40 cmH_2_O (approximately 29.41 mmHg). Avoiding luminal overexpansion will decrease the risk of potential induction of suture tract leakage (43). Accordingly, the intraluminal leakage pressure value at 30 mmHg was set in the present study. One leakage in the figure-of-eight suture group and two in the interrupted suture group occurred at the suture hole or anastomotic line. Other research suggests that the leakage may be linked to the needle size or a technique related to tissue tearing (44). The first stitch in the asymmetric figure-of-eight suture technique included whole layers, and the second stitch covered three layers except the mucosal layer. Furthermore, the two stitches were not at a horizontal level, which may lead to a shorter intersuture distance and tension-free while tying the knot, resulting in a secure anastomosis. This alternative suture technique might minimize the tension and avoid cutting through the fragile tissue (45). Anti-tension of the anastomosis caused by suturing the submucosa twice might improve the security of the anastomosis. Early and tight mucosal apposition may protect the bowel anastomosis from luminal content stimulation and potential infection (46, 47). Although this single-layer suture technic for intestinal anastomosis was feasible, the knot must be carefully tied with adequate strength to avoid any possible tissue strangulation at the anastomotic site (30), and an additional Lembert's suture should be placed if needed (48).

## Limitations

This study has several limitations. First, it is an *in vitro* experiment with small sample size. Second, an *in vitro* study could not simulate the pathophysiological process of intestinal anastomosis, including intra-abdominal adhesion formation. Although the new synthetic absorbable sutures seem to generate less adhesion response, steps such as avoiding excessive suture materials could play a role in preventing adhesion formation (49–51). The effects of suture material exposure on adhesion formation need to be clarified in animal models. Third, all cadaveric ileum specimens were harvested from one pig, which might not cover the intestine specimens from the general population of pigs.

In conclusion, the preliminary *in vitro* study showed that the single-layer asymmetric figure-of-eight suture anastomosis technique was feasible and low-cost, but essentially it is a safe pattern for intestinal anastomosis, which presented less leakage than the single-layer interrupted suture technique.

## Data Availability

The original contributions presented in the study are included in the article/Supplementary Material, further inquiries can be directed to the corresponding authors.
